# Assessment of the Vaccination Program against Cystic Echinococcosis in Sheep in the Pehuenche Community of Central Chile

**DOI:** 10.3390/ani12060679

**Published:** 2022-03-08

**Authors:** Paula Gädicke, David Heath, Angela Medina-Brunet, María Carolina Siva-de la Fuente, Hellen Espinoza-Rojas, Carmen Villaguala-Pacheco, Makarena Rubilar, Carolina Cerda, Manuel Quezada, Daniela Rojas, AnaLía Henríquez, Marco Loyola, Carlos Landaeta-Aqueveque

**Affiliations:** 1 Facultad de Ciencias Veterinarias, Universidad de Concepción, Vicente Méndez 595, Chillán 3812120, Chile; pgadicke@udec.cl (P.G.); angela.medinabrunet@gmail.com (A.M.-B.); hellen.constanza@gmail.com (H.E.-R.); cvillaguala@udec.cl (C.V.-P.); makrubilar@udec.cl (M.R.); carolinaacp.vet@gmail.com (C.C.); mquezad@udec.cl (M.Q.); drojasm@udec.cl (D.R.); 2 Wallaceville Animal Research Centre, AgResearch, P.O. Box 40063, Upper Hutt 6007, New Zealand; heathdd@gmail.com; 3 Facultad de Ciencias Veterinarias, Universidad Austral, Campus Isla Teja s/n, Valdivia 5090000, Chile; silva.delafuente@gmail.com; 4 Facultad de Medicina Veterinaria, Universidad San Sebastián, Lientur 1457, Concepción 4081375, Chile; alhenriquezh@gmail.com (A.H.); marco.loyola@uss.cl (M.L.)

**Keywords:** *Echinococcus*, Eg95, vaccine, ovine, hydatid cyst, echinococcosis control, Indigenous people, zoonosis

## Abstract

**Simple Summary:**

Cystic echinococcosis is a parasitic disease affecting humans; in Chile, it uses sheep and dogs as its main hosts. The Eg95 vaccine has been developed with the aim of controlling ovine infection. Here, we present the results of a 3-year control program in the Alto Biobío commune in central Chile. The program tried to provide a first dose at 2 months of age, a booster 1 month later, and yearly vaccination. Given the difficult land work, important delays in the vaccinations were recorded, and many animals did not receive the first booster. Dog deworming was not included in the program. The main results of the program were that after vaccination, the proportion of large and fertile cysts was lower than before; however, the proportion of infected sheep had not reduced. In addition, the lower age at first dose and the administration of the second dose 1 month after the first were associated with greater protection. Hence, the results suggest that vaccination was not effective against the infection of sheep, but it was effective against the development of cysts; thus, cysts are less infective for dogs. This could favor disease control by cutting the cycle.

**Abstract:**

Echinococcosis is a neglected zoonosis that uses dogs and sheep as its main hosts in Chile. The Eg95 vaccine against sheep infection has been included in some control programs. Here, we assess the efficacy of the vaccination program in the hyperendemic Alto Biobío commune after 3 years of execution. Fisher’s test and generalized linear models were used in the assessment. The program tried to offer a first dose at 2 months of age, a booster 1 month later, and yearly vaccination. Given logistic difficulties, important delays in vaccination occurred, and most animals did not receive the first booster. Dog deworming was not included in the program. Likely due to the aforementioned factors, the overall frequency of infection was not lower, but the proportion of large (>5 mm) cysts and fertile cysts was smaller after the program. The frequency of infection and/or the number of cysts were lower when the age at first dose was younger and the first booster was administered 1 month after the first dose. The results suggest that vaccination affects both cyst development after the larvae reach the target organs, as well as the development of the protoscolex once the cysts start developing.

## 1. Introduction

Cystic echinococcosis (CE) is a parasitic zoonosis with a worldwide distribution and is caused by cestodes of the *Echinococcus granulosus* sensu lato (s.l.). complex. Canids are the definitive host and become infected after the consumption of raw or undercooked metacestodes. Several mammals, including humans, host the metacestode, known as a hydatid cyst, and become infected after consuming eggs released in dog feces [1,2]. Several genotypes and species have been described within *E. granulosus* s.l., in variable associations with different intermediate hosts, with the genotype G1 (*E. granulosus* s.s.) being the most frequently reported worldwide, associated with lung and hepatic cysts in sheep, goats and humans [1,3,4].

CE is considered by the World Health Organization (WHO) to be a neglected disease; it causes not only deaths, but also several aftereffects and disability in survivors, which means there are important human costs [5]. This disease may have economic costs for public health systems, but the economic costs for livestock production can be even higher than those associated with human health [6,7]. CE control was historically achieved by controlling the free-roaming dog population, deworming dogs, performing veterinary inspection in the abattoirs, and improving practices in rural communities to prevent the domestic slaughter of livestock, the consumption of raw viscera by dogs and the contamination of food and water with dog feces [8]. However, the vaccination of sheep is a new approach that has been available since the last couple of decades. The currently available vaccine uses the Eg95 protein to boost immunity [9]. Since the resistance to *Echinococcus* eggs in the environment is considerable, due to the viability of oncospheres even after 3 years, implementation of a control program primarily based on the deworming of dogs is a long-term challenge. Hence, the availability of a vaccine that stops the cycle could allow the program to achieve its goals more rapidly.

Several studies have assessed the efficacy of the Eg95 vaccine with varying results, largely dependent on the type of study conducted. Thus, this vaccine has shown good results in pilot experimental studies in Australia and Argentina that examined the effects of two vaccinations and an exposition 30 days after; this approach was associated with an efficacy rate of over 80%. Pilot experimental studies also shown positive results in Iran, China and Chile [10,11,12]; however, the observed efficacy in field studies was lower. For instance, in Río Negro, Argentina, after a 5-year protocol consisting of three vaccinations that were administered in the first year (at 1, 2 and 12 months of age), the prevalence decreased from 56.3% to 21.0% [13].

In Chile, human CE is more frequently present in the central and southern regions [14,15], although high variations have been observed within regions and between communes [16,17,18]. Knowledge of this disease is low in central Chile, and high death rates are reported in the Alto Biobío commune. This commune is compounded by a high percentage of Pehuenche (Indigenous—“pueblos originarios”) people, who engage in agricultural economic activities that are focused on breeding sheep and goat flocks; this population is also characterized by low economic incomes, which are factors that favor the presence of CE [17,19]. Given the above, a vaccination program for sheep was initiated in 2016. The aims of this study were to describe the occurrence of hydatid cysts in sheep before the implementation of the vaccination program and to epidemiologically assess the efficacy of this program after 3 years of execution.

## 2. Materials and Methods

The Alto Biobío commune is located in the eastern Biobío region, in the Los Andes Mountain Range. The 2017 census indicated that a total of 5923 people live in the Alto Biobío commune, with 86% belonging to the Pehuenche community; the primary wage earner has an average education of 6.5 years [20]. As of 2017, the Alto Biobío commune had among the highest poverty rates in Chile [21]. The latest agricultural census with published results was performed in 2007 and reported that in this commune, 8137 sheep were distributed across 454 farms, with an average of 18 animals per flock and 254 ha of surface, but only 2.3 ha of grasslands on average [22]. Livestock production is characterized by seasonal migrations, where the highlands are used during summer and autumn and lowlands used in winter and spring. Although there are no published estimations of the dog population during this study, an average of at least one dog per farm was seen during fieldwork. The Ralco National Reserve is located within the commune, and although no reports of native mammals have been published, the commune is within the distribution of chilla foxes (*Lycalopex griseus*), which have reportedly been infected with *E. granulosus* s.l. [23].

**Figure 1:**
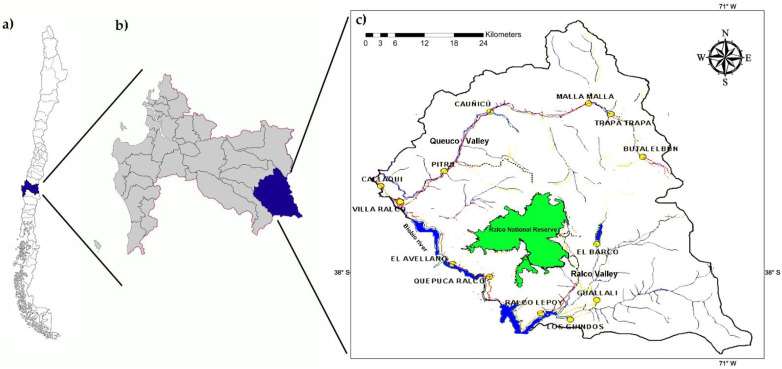
(**a**) Map of Chile with administrative regions. (**b**) Map of the Biobío region and its location in Chile. (**c**) Map of the Alto Biobío commune, with its location in the Biobío region; the main rivers and localities that participated in assessments of the Eg95 vaccination program are shown.

There are three main rivers cross the commune: The Queuco river begins at the east of the commune, north of the Copahue volcano, drawing an inverted U to the west near the northern limits of the commune, until it flows into the Biobío River. The Ralco River begins between the Callaqui and the Copahue volcanoes and moves straight to the south-west, where it flows into the Biobío River. The Biobío River marks the southwesternmost limit of the commune between the discharges of the Queuco and Ralco rivers (Figure 1). People are distributed in two major sections of the commune: the Queuco valley (which includes the Queuco river and its tributaries) and the Biobío valley (which includes the Biobío River, the Ralco river and its tributaries). Several communities are distributed along these two valleys. Butalelbún, Trapa-Trapa, Malla-Malla, Cauñicu and Pitril are the communities located in the Queuco valley that participated in the program. Further, Guallalí, El Barco, Ralco-Lepoy, El Avellano, Los Guindos and Quepuca-Ralco are located in the Biobío valley. Callaqui is located where the Queuco River discharges into the Biobío River. Given their short distance and similarities, Trapa-Trapa and Butalelbún were grouped into a single cluster for this study and are henceforth referred to as the Trapa-Trapa cluster.

According to information provided by the Agricultural and Cattle Service (referred to by the Spanish acronym, “SAG”), the program started in late spring of 2016 with the first administration of the Providean Hidatil EG95^®^ vaccine among all sheep flocks; the vaccine was administered as soon as possible after 2 months of age. During the program, an attempt was made to administer a second vaccine 1 month after the first, followed by yearly administrations. However, most lambs received the first dose after 3 months of age due to fieldwork difficulties and seasonal migrations. As such, most animals did not receive the 1-month booster. In addition, there was a 6-month delay in summer 2017 given that the Providean Hidatec EG95^®^ vaccine (Tecnovax, Buenos Aires, Argentina) was being changed to a lyophilized version. This change was made given that unwanted tissue reactions to the oil-formulated Hidatil EG95 developed in animals after the first administration. This delay led to a longer exposure time of lambs to infection. The program only considered ovine vaccination. No deworming of dog or sheep was transversally performed across the commune.

Two assessments of ovine CE occurrence were performed: the first took place during the spring of 2016, directly before the start of the vaccination program, and the second was performed in January 2020, directly before the summer–autumn migration in most localities. Following Vallejo et al. [24], in order to calculate a proportion close to 10.9% [25] with a 95% confidence and a 5% of accepted error, at least 194 individuals had to be examined. Efficacy was assessed based on the comparison of both studies. The first assessment considered 223 sheep from nine localities, and the second considered 200 sheep from ten localities (see Table 1 for details on the animals analyzed by locality in 2016 and 2020). In both assessments, the liver and lungs of animals were examined macroscopically to determine the presence of hydatid cysts. Viscera were obtained and were joined to the head by the trachea in order to link them using individual ear tag numbers, which featured traceable information on the time of each vaccination. Viscera were kept with icepacks in adequate containers and were examined the next day after the animals were slaughtered. Some large cysts (>5 mm) were examined to assess fertility. Macroscopic visualization of the germinal layer and microscopic visualization of the protoscolex in the hydatid liquid were the criteria that were used to confirm the diagnosis of a fertile large cyst. The presence of the germinal layer and absence of the protoscolex confirmed the diagnosis of an infertile cyst. Histopathological examinations were performed to confirm the diagnosis in small cysts (<5 mm), verifying the presence of the germinative, laminar and adventitial layers. This examination was performed using cuts of tissues with lesions, which were processed in an automatic processor; 4 μm sections were stained with hematoxylin and eosin (H&E) stain.

The frequencies of infection with any hydatid cyst and with infecting cysts are presented in terms of percentage with 95% confidence intervals (CIs). The number of animals with fertile cysts in the whole sample was estimated as an extrapolation of the proportion of the animals with fertile cysts among those that were examined.

Vaccination was assessed in two ways: using comparisons of the frequency of cysts between 2016 and 2020 and comparisons of the fertility of cysts between 2016 and 2020. Fisher’s exact test was used for those comparisons, with a *p* < 0.05 significance level.

Multifactorial linear generalized models (LGM) with a binomial probability distribution and logit link function were used to assess the association between the presence/absence of cysts and the independent variables: age at first administration, type of vaccine (oil or lyophilized), number of vaccinations administered until the examination, and administration or not of a booster 1 month after first administration. LGM with a negative binomial probability distribution and log-link function were used to assess the association between the abundance of large cysts and the same independent variables. The analysis began with the full model, and then the less significant variable was removed in each step. Given the independence of the variables, no interactions were assessed. The likelihood ratio (LR) test criterion was used to decide whether to remove a variable. In both cases—presence/absence and abundance—the final model was selected when the removal of any variable implied a significant loss of likelihood. Given that the removal of a variable implies the acceptance of the null hypothesis of the LR test, the significance level for the LR tests was *p* < 0.1. See Table S1 in the supporting information for data included in the LGM. The association of age, as well as the association of the locality with the presence/absence of a hydatid cyst, were assessed with a simple LGM with a binomial probability distribution and logit link function for each. In the case of locality, Los Guindos served as the basal comparison category, and the other localities were considered as dummy variables. The correlation between independent variables was analyzed with Spearman rank correlation tests. Analyses were performed with Stata/BE 17.0 (StataCorp LLC, College Station, TX, USA).

The study was approved by the Comité de Bioética of the Facultad de Ciencias Ve-terinarias of the Universidad de Concepción (CBE-27-2021).

## 3. Results

### 3.1. Spring 2016 (Pre-Vaccination Sampling)

Guallalí, El Barco, Ralco Lepoy, Butalelbún and Trapa-Trapa were the localities with the largest number of examined animals (Table 1).

The localities with the highest frequency of CE in adult animals (>2 years) were Trapa-Trapa, Butalelbún, Ralco-Lepoy and El Barco, where more than 60% of animals were infected. Conversely, Los Guindos showed a frequency that was significantly lower than in every locality but Cauñicú and El Avellano (positive coefficients in all cases; *p* = 0.2 in Cauñicú, not analyzed with El Avellano, and *p* < 0.024 in the other cases; parameters were omitted). Table 1 shows the frequencies by localities; however, in order to compare these samples with the 2020 sample, only animals 2–4 years of age were considered. As previously mentioned, given the proximity of the Butalelbún and Trapa-Trapa localities, they were considered as a single geographic unit: the Trapa-Trapa cluster.

Most cysts were large (608 out of 928), and 190 of them were examined to assess fertility. In all, 60% (*n* = 114) of all cysts were fertile.

The overall frequency of CE was 45.29% (considering all animals) and showed a significant increase in older animals (coefficient =0.53, Z = 4.87, *p* < 0.01; Table 2); the age of 10 sheep could not be estimated.

### 3.2. January 2020 (Post-Vaccination Sampling)

**Figure 2:**
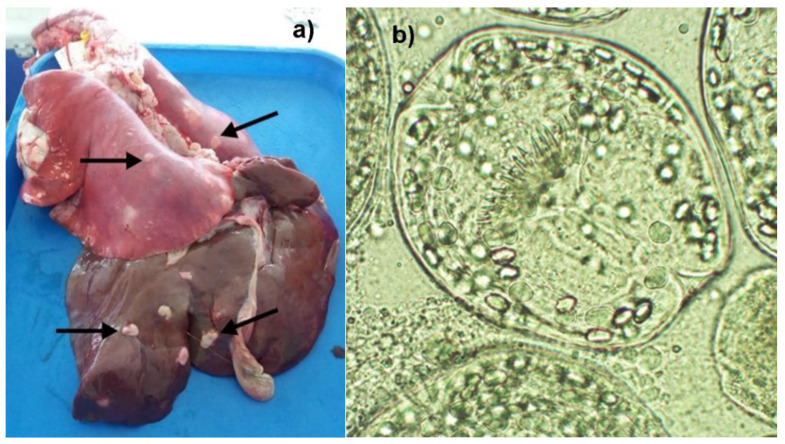
(**a**) Hydatid cysts in the lung and liver of sheep (arrow). (**b**) Protoscolex of *Echinococcus granulosus* s.l. extracted from a large hydatid cyst. Alto Biobío, 2020.

Overall, 2774 cystic structures were found, 64.74% of which were smaller than 5 mm. Only 17.1% of examined large cysts were fertile. The overall frequency of hydatid cyst occurrence was 55% in sheep, and the frequency of fertile cyst occurrence was 8% in animals. Figure 2 shows larger cysts and the protoscolex found in fertile cysts. The highest frequencies of sheep with fertile cysts were found in Guallalí, Malla-Malla and the Trapa-Trapa cluster (Table 1).

**Figure 3:**
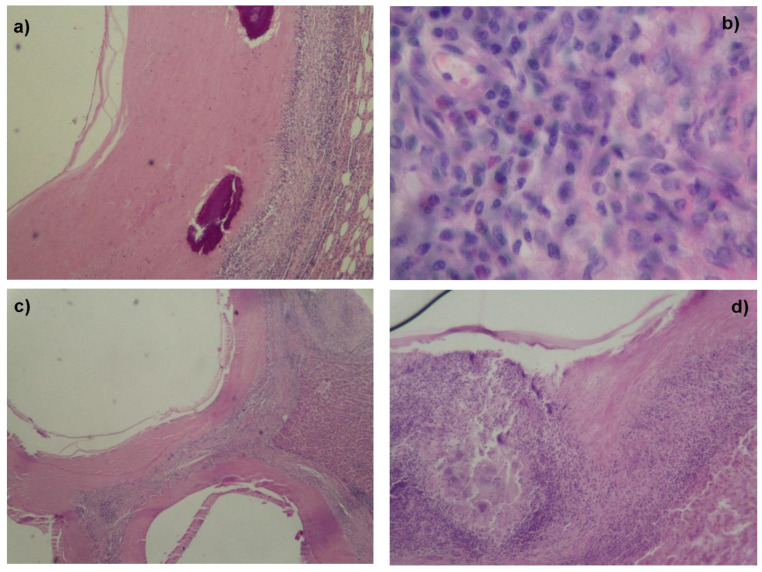
Small hydatid cyst extracted from sheep liver and lungs (hematoxylin-eosin (H&E) stain). (**a**) The three characteristic layers of the cyst and amorphous deposits stained dark violet with H&E, concordant with calcium deposits. (**b**) Granulomatous inflammation with the presence of multinucleated giant cells and some eosinophilic polymorphonuclear leukocytes. (**c**) Miliary pattern of multiple coalescing cysts with a peripheral granulomatous inflammatory response. (**d**) Granulomas with detritus in the center and a peripheral adventitial layer. Alto Biobío, 2020.

The histological examination of all small cysts obtained from both 2016 and 2020 samplings (*n* = 40) indicated that only one did not correspond to a hydatid cyst. Histologically, in both the liver and lung, structures smaller than 5 mm corresponded to cysts that had their three histological layers preserved, and outside the adventitial layer, there was a severe mononuclear inflammatory infiltrate characterized by plasma cells, lymphocytes and macrophages (Figure 3a). In some of the evaluated structures, granulomatous inflammation was observed with the presence of multinucleated giant cells and some eosinophilic polymorphonuclear leukocytes (Figure 3b). Many of the observed structures presented in a miliary pattern in which multiple coalescing cysts with a peripheral granulomatous inflammatory response were found (Figure 3c). Some of the observed structures corresponded to granulomas with detritus in the center and a peripheral adventitial layer infiltrated by the same inflammatory response observed in cysts that presented the three layers of tissue (Figure 3d). In some of the evaluated structures, amorphous, dark-violet H&E-stained deposits were observed, concordant with calcium deposits (Figure 3a).

With the aim of comparing equivalent populations from 2016 and 2020, only 139 sheep that were 2–4 years old from the 2016 sampling were used in the comparative analysis. It was noted that the frequency of infection in 2020 was similar to that in 2016 (Fisher’s exact test: *p* = 0.15). However, the proportion of large cysts across all cysts was lower in 2020 (35.26%) than in 2016 (65.52%; *p* < 0.001). As well, among large cysts, the proportion of fertile cysts was lower in 2020 (17.1%) than in 2016 (60%; Fisher’s exact test: *p* < 0.01). When extrapolating the fertility rate, it was estimated that 28.1% of all sheep examined in 2016 were infected with fertile cysts. This proportion was significantly lower in 2020 (8%; *p* < 0.01).

The selected LGM included booster administration (1 month after the first dose) and age at first dose as variables associated with the presence/absence of fertile hosts. The likelihood of being infected with fertile cysts decreased when the booster was administered (versus not administered), and it increased in association with older age at first dose. Conversely, the selected LGM included booster administration and the number of vaccines received by the animal, insofar as the number of cysts was smaller if the booster was administered and larger if the number of vaccines received by the animals increased (see Table 3 for details of the models).

The removal of a variable implied a significant loss of likelihood (LR tests: *p* < 0.03 in all cases). The number of received vaccines was significantly correlated with the amount of time in the program (Rho = 0.34; *p* < 0.01); however, these variables were kept in the model given the LR test results.

## 4. Discussion

The variation in prevalence among localities in the first assessment could have been due to local factors that were not measured in the study, such as the knowledge and practices of animal owners, which were risk factors that have been reported previously in Chile [25]. These results could also be due to the density of livestock, which were reportedly associated with infection rates elsewhere [26,27,28]. Another study aimed to assess the knowledge and practices surrounding infection during 2020, but the COVID-19 pandemic made it impossible. With respect to sheep density, since the density varies between farms within localities, and given that the seasonal migrations led to variations in density between seasons (as this variation is not necessarily the same across localities), no valid conclusions can be drawn about its association with the presence or abundance of cysts.

This program only included sheep vaccination. To the best of our knowledge, no deworming programs that target the entire dog population were implemented in the commune prior to or during the vaccination program. Only some dogs owned by people diagnosed with cystic echinococcosis were dewormed by health services. Hence, it is likely that almost all areas will have large numbers of *E. granulosus* s.l. eggs that young lambs may ingest, even at 2 months of age.

Due to the conditions of the program—specifically, that vaccine administration was conducted among all sheep flocks—it was not possible to have a control group for comparison (i.e., a group of unvaccinated sheep bred together with vaccinated ones). Therefore, it was not possible to draw conclusions about the similarity in prevalence rates between both assessments. However, some results enabled the comparison of the 2016 and 2020 findings. For instance, it was possible to state that all sheep with hydatid cysts were exposed to oncosphere larva, and that the differences in cyst fertility rates between the 2016 and 2020 samples could have been caused by the administration of the Eg95 vaccine. Thus, the proportion of large cysts across all cysts was lower in the vaccinated sample from 2020 than in the 2016 sample. In addition, in the 2020 sample, both the proportion of fertile cysts across large cysts and the frequency of sheep infected with fertile cysts were significantly lower than in the unvaccinated 2016 sample. These differences suggest that vaccine administration could reduce the infection rate in dogs that feed on these cysts, favoring the control of this disease by cutting the cycle. Previous results provided evidence to suggest that better results in sheep infection rates were obtained when dog deworming was included in the program [29].

The Eg95 protein is not only expressed in the oncosphere, it is also expressed in other moments of the cycle, such as in the protoscolex and adult intestinal stage. However, the study by Zhang et al. [30], which observed these expressions, did not assess the expression of this protein in the cyst wall during the start of its development. Considering that the difficulties experienced in field management when conducting the vaccination program led to delays in first-dose and subsequent-dose vaccinations, our results—particularly the association between the age at first dose and the presence of a cyst—suggest that there is an effect of the vaccine at the beginning of cyst development. However, there is a lack of evidence to suggest that the vaccine could prevent cyst development altogether. New studies testing the presence of the Eg95 protein in cysts during their beginnings are necessary to explain this situation. Similarly, the lower frequency of the protoscolex in large cysts after vaccination (2020) compared with the data before vaccination (2016) suggests that the development of the protoscolex within the cysts is affected by the vaccine. This is supported by the negative association of the presence and abundance of fertile cysts and the one-month-later booster administration. It is also supported by the fact that the host’s antibodies, particularly immunoglobulin (Ig)G, are able to enter into the cyst, which is associated with lower fertility of the cyst [31,32]. Given that immunity due to the vaccine has been measured in IgG concentrations [33,34], the results suggest that the vaccine affects cyst fertility. In this way, the higher proportion of infertile cysts could be due to the late administration of the first vaccine, late administration of the first booster, or a deficient immune response of the sheep to some of these doses. Thus, subsequent doses could have been responsible for the infertility of large cysts. Again, the presence of the Eg95 protein in these cysts must be tested to draw stronger conclusions. The aforementioned findings suggest that yearly boosters are useful when there is no certainty of achieving the vaccination of all sheep in a single campaign/year.

The effect of the vaccine on genotypes other than G1 is not clear. The molecular composition of the Eg95 protein varies between some genotypes, as it is different between the G1–G2 and G6–G7 genotypes [35,36]. Cysts were not genotyped in the present study; however, the high fertility seen in the 2016 sample suggests that G1 is the dominating genotype. Despite this, it is not possible to dismiss the likelihood that another genotype is also circulating. Other genotypes reported in Chile are G3, G5 and G6 [37,38,39], and they have a lower affinity for sheep [40,41]. Hence, if these genotypes were present in the Alto Biobío commune, that lower affinity could explain the presence of small or infertile cysts in sheep. Further molecular studies are necessary to test this hypothesis.

The better protection of the oil vaccination (lower number of cysts) contrasts with the unwanted tissue reactions, which should be considered when making decisions. Finally, the positive association of the number of received vaccines and the time in the program with the number of cysts is explained by the fact that these variables were positively associated with age, and that age is positively associated with the abundance of cysts. It should not be understood as a lower protection when more vaccines are administered.

## 5. Conclusions

The Alto Biobío commune has an uneven distribution in terms of the frequency of hydatid cyst occurrence in sheep. Administration of the Eg95 vaccine in ovine livestock in the Alto Biobío commune has been a challenge. Although the occurrence of cysts was not less frequent, as expected, the experiences associated with this program suggest that vaccination has unexpected effects that warrant additional tests. The results reported herein suggest that vaccination affects the development of cysts after the larvae arrive at the target organs, and it also affects the development of the protoscolex once the cysts start developing.

## Figures and Tables

**Table 1:** Frequency of occurrence (95% confidence interval (CI)) of hydatid cysts in lung–liver samples of sheep of 2–4 years of age by locality and year of sampling, in the Alto Biobío commune, Chile.

Locality	2016		2020		
	Number of Infected/Examined Animals	Frequency % (95% CI)	Number of Infected/Examined Animals	Frequency % (95% CI)	Frequency of Fertile Hydatid Cysts % (95% CI)
Trapa-Trapa cluster	18/28	64.29 (16.1–82.52)	58/91	63.7 (53.7–73.7)	8.8 (2.9–14.7)
Malla-Malla	0	–	11/19	57.9 (34.9–80.8)	10.53 (0–24.8)
Cauñicú	1/1	100 (5–100)	1/5	20 (0–59.4)	0 (0–45.1)
Pitril	0	–	2/5	40 (0–88.3)	0 (0–45.1)
Callaqui	0	–	0/4	0 (0–52.7)	0 (0–52.7)
El Avellano	0/1	0 (0–95)	0	–	–
Quepuca Ralco	3/5	60 (11.6–100)	5/16	31.3 (7.6–54.9)	6.3% (0–18.6)
Ralco Lepoy	15/28	53.6 (35.6–72.5)	18/27	66.7 (48.4–84.9)	3.7 (0–11.0)
Los Guindos	2/21	9.5 (0–22.5)	0	–	–
Guallalí	13/29	44.8 (26.2–63.4)	7/18	38.9 (15.6–62.2)	16.7 (0–34.5)
El Barco	13/26	50 (30.2–69.8)	8/15	53.3 (27.0–79.6)	6.7 (0–19.8)
Overall	65/139	46.8 (38.4–55.2)	110/200	55 (48.0–62.0)	8 (4.2–11.8)

**Table 2:** Frequency (%) of infection and mean abundance of hydatid cysts in lung–liver samples of sheep from the Alto Biobío commune, Chile, by age (year) in spring 2016.

Age	Sample Size	Frequency of Infection	Mean Abundance of Cysts
1	39	23.08	1.85
2	52	36.53	1.08
3	51	47.06	2.52
4	36	61.11	5.41
5	22	77.27	17.55
6	11	72.73	6.73
7	1	1.00	3.00
8	1	1.00	13.00

**Table 3:** Parameters of selected linear generalized models for the presence/absence and abundance of fertile hydatid cysts after the vaccination program in the Alto Biobío commune, Chile. The binomial probability distribution and logit-link function were used for the presence/absence model, and the negative binomial probability distribution and log-link function were used for the abundance model.

Presence/Absence of Fertile Cysts *	Coefficient	Standard Error	*p* Value	95% Confidence Interval
Age at first dose	0.276	0.123	0.025	0.035–0.518
One-month-after booster	−1.578	0.796	0.047	−3.138–−0.018
Abundance of Fertile Cysts *	**Coefficient**	**Standard Error**	***p* Value**	**95% Confidence Interval**
Lyophilized vaccine (versus oil vaccine)	1.006	0.326	0.002	0.368–1.644
One-month-after booster	−0.539	0.231	0.019	−0.992–−0.087
Number of received vaccines	0.745	0.260	0.004	0.236–1.254
Time in program	0.002	0.001	<0.001	0.001–0.004

* Bold fonts indicate the models.

## Data Availability

The data presented in this study are available in Table S1 of the supplementary material.

## Supplementary Materials

The following supporting information can be downloaded at https://www.mdpi.com/article/10.3390/ani12060679/s1: Table S1: Raw data of sheep examined for the presence and abundance of hydatid cysts in the Alto Biobío commune.

## References

[1] Nakao M., Lavikainen A., Yanagida T., Ito A. (2013). Phylogenetic systematics of the genus *Echinococcus* (Cestoda: Taeniidae). Int. J. Parasitol..

[2] Woolsey I.D., Miller A.L. (2021). *Echinococcus granulosus* sensu lato and *Echinococcus multilocularis*: A review. Res. Vet. Sci..

[3] Alvarez Rojas C.A., Romig T., Lightowlers M.W. (2014). *Echinococcus granulosus* sensu lato genotypes infecting humans—Review of current knowledge. Int. J. Parasitol..

[4] Romig T., Deplazes P., Jenkins D., Giraudoux P., Massolo A., Craig P.S., Wassermann M., Takahashi K., De La Rue M. (2017). Ecology and life cycle patterns of *Echinococcus* species. Adv. Parasitol..

[5] WHO Echinococcosis. https://www.who.int/news-room/fact-sheets/detail/echinococcosis.

[6] Bingham G.M., Hererro E., Budke C.M., Norby B., Uchiumi L., Larrieu E., Del Carpio M., Mujica G., Mercapide C., Salvitti J.C. (2016). The economic Iimpact of cystic echinococcosis in Rio Negro Province, Argentina. Am. J. Trop. Med. Hyg..

[7] Moro P.L., Budke C.M., Schantz P.M., Vasquez J., Santivañez S.J., Villavicencio J. (2011). Economic impact of cystic echinococcosis in Peru. PLoS Negl. Trop. Dis..

[8] Craig P., Larrieu E. (2006). Control of Cystic Echinococcosis/Hydatidosis: 1863–2002. Adv. Parasitol..

[9] Lightowlers M.W., Lawrence S.B., Gauci C.G., Young J., Ralston M.J., Maas D., Heath D.D. (1996). Vaccination against hydatidosis using a defined recombinant antigen. Parasite Immunol..

[10] Heath D.D., Jensen O., Lightowlers M.W. (2003). Progress in control of hydatidosis using vaccination—A review of formulation and delivery of the vaccine and recommendations for practical use in control programmes. Acta Trop..

[11] Paykari H., Heath D.D., Dalimi A.H., Karimi G.R., Motamedi G.R. (2008). Experimental vaccination of sheep against hydatid cyst using EG95 recombinant vaccine. Arch. Razi Inst..

[12] Vivallo Cuevas I.O. (2004). Evaluación de la Vacuna Eg95 Contra Hidatidosis, en Ovinos. Ph.D. Thesis.

[13] Larrieu E., Mujica G., Gauci C.G., Vizcaychipi K., Seleiman M., Herrero E., Labanchi J.L., Araya D., Sepúlveda L., Grizmado C. (2015). Pilot Field Trial of the EG95 Vaccine Against Ovine Cystic Echinococcosis in Rio Negro, Argentina: Second Study of Impact. PLoS Negl. Trop. Dis..

[14] Cortés S., Valle C. (2010). Hidatidosis humana: Generalidades y situación epidemiológica en Chile según egresos hospitalarios y notificación obligatoria entre los años 2001 y 2005. Rev. Chil. Infectol..

[15] Colombe S., Togami E., Gelaw F., Antillon M., Fuentes R., Weinberger D.M. (2017). Trends and correlates of cystic echinococcosis in Chile: 2001–2012. PLoS Negl. Trop. Dis..

[16] Soto-Aguilar A., Junod T., Campillay M., Acosta-Jamett G., Landaeta-Aqueveque C. (2017). Análisis de la hidatidosis humana en la Región de Coquimbo entre los años 2008 y 2012. Rev. Méd. Chile.

[17] Medina N., Riquelme N., Rodríguez J., Aguirre O., Ayala S., Canals M. (2019). Distribución y factores de riesgo de hidatidosis en la Región del Libertador Bernardo O’Higgins entre 2010 y 2016. Rev. Chil. Infectol..

[18] Medina N., Martínez P., Ayala S., Canals M. (2021). Distribución y factores de riesgo de equinococosis quística humana en Aysén 2010–2016. Rev. Chil. Infectol..

[19] Khan A., Ahmed H., Simsek S., Gondal M.A., Afzal M.S., Irum S., Muhammad I., Mansur H., Fatima A., Ali M.S. (2019). Poverty-associated emerging infection of cystic echinococcosis in population of Northern Pakistan: A hospital based study. Trop. Biomed..

[20] Resultados Censo 2017. http://resultados.censo2017.cl/Region?R=R08.

[21] Alto Biobío, Reporte Comunal. https://www.bcn.cl/siit/reportescomunales/comunas_v.html?anno=2020&idcom=8314.

[22] Censo Agropecuario. https://www.ine.cl/estadisticas/economia/agricultura-agroindustria-y-pesca/censos-agropecuarios.

[23] Acosta-Jamett G., Cleaveland S., de C Bronsvoort B.M., Cunningham A.A., Bradshaw H., Craig P.S. (2015). *Echinococcus granulosus* infection in foxes in Coquimbo District, Chile. Arch. Med. Vet..

[24] Vallejo A., Muniesa A., Ferreira C., Blas I.d. (2013). New method to estimate the sample size for calculation of a proportion assuming binomial distribution. Res. Vet. Sci..

[25] Acosta-Jamett G., Cleaveland S., Cunningham A.A., Bronsvoort B.M.d., Craig P.S. (2010). *Echinococcus granulosus* infection in humans and livestock in the Coquimbo region, north-central Chile. Vet. Parasitol..

[26] Avila H.G., Santos G.B., Cucher M.A., Macchiaroli N., Pérez M.G., Baldi G., Jensen O., Pérez V., López R., Negro P. (2017). Implementation of new tools in molecular epidemiology studies of *Echinococcus granulosus* sensu lato in South America. Parasitol. Int..

[27] Chaâbane-Banaoues R., Oudni-M’Rad M., Cabaret J., M’Rad S., Mezhoud H., Babba H. (2015). Infection of dogs with *Echinococcus granulosus*: Causes and consequences in an hyperendemic area. Parasites Vectors.

[28] Ghatee M.A., Nikaein K., Taylor W.R., Karamian M., Alidadi H., Kanannejad Z., Sehatpour F., Zarei F., Pouladfar G. (2020). Environmental, climatic and host population risk factors of human cystic echinococcosis in southwest of Iran. BMC Public Health.

[29] Larrieu E., Mujica G., Araya D., Labanchi J.L., Arezo M., Herrero E., Santillán G., Vizcaychipi K., Uchiumi L., Salvitti J.C. (2019). Pilot field trial of the EG95 vaccine against ovine cystic echinococcosis in Rio Negro, Argentina: 8 years of work. Acta Trop..

[30] Zhang W.B., Li J., You H., Zhang Z.Z., Turson G., Loukas A., McManus D.P. (2003). Short report: *Echinococcus granulosus* from Xinjiang, PR China: cDNAS encoding the EG95 vaccine antigen are expressed in different life cycle stages and are conserved in the oncosphere. Am. J. Trop. Med. Hyg..

[31] Paredes R., Godoy P., Rodríguez B., García M.P., Cabezón C., Cabrera G., Jiménez V., Hellman U., Sáenz L., Ferreira A. (2011). Bovine (*Bos taurus*) humoral immune response against *Echinococcus granulosus* and hydatid cyst infertility. J. Cell. Biochem..

[32] Riesle S., García M.P., Hidalgo C., Galanti N., Saenz L., Paredes R. (2014). Bovine IgG subclasses and fertility of *Echinococcus granulosus* hydatid cysts. Vet. Parasitol..

[33] Larrieu E., Poggio T.V., Mujica G., Gauci C.G., Labanchi J.L., Herrero E., Araya D., Grizmado C., Calabro A., Talmon G. (2017). Pilot field trial of the EG95 vaccine against ovine cystic echinococcosis in Rio Negro, Argentina: Humoral response to the vaccine. Parasitol. Int..

[34] Heath D.D., Koolaard J. (2012). Serological monitoring of protection of sheep against *Echinococcus granulosus* induced by the EG95 vaccine. Parasite Immunol..

[35] Alvarez Rojas C.A., Gauci C.G., Lightowlers M.W. (2013). Antigenic differences between the EG95-related proteins from *Echinococcus granulosus* G1 and G6 genotypes: Implications for vaccination. Parasite Immunol..

[36] Chow C., Gauci C.G., Vural G., Jenkins D.J., Heath D.D., Rosenzvit M.C., Harandi M.F., Lightowlers M.W. (2008). *Echinococcus granulosus*: Variability of the host-protective EG95 vaccine antigen in G6 and G7 genotypic variants. Exp. Parasitol..

[37] Manterola C., Benavente F., Melo A., Vial M., Roa J.C. (2008). Description of *Echinococcus granulosus* genotypes in human hydatidosis in a region of southern Chile. Parasitol. Int..

[38] Espinoza S., Salas A.M., Vargas A., Freire V., Diaz E., Sánchez G., Venegas J. (2014). Detection of the G3 genotype of *Echinococcus granulosus* from hydatid cysts of Chilean cattle using COX1and ND1mitochondrial markers. Parasitol. Res..

[39] Corrêa F., Stoore C., Horlacher P., Jiménez M., Hidalgo C., Alvarez Rojas C.A., Figueiredo Barros G., Bunselmeyer Ferreira H., Hernández M., Cabrera G. (2018). First description of *Echinococcus ortleppi* and cystic echinococcosis infection status in Chile. PLoS ONE.

[40] Nakao M., McManus D.P., Schantz P.M., Craig P.S., Ito A. (2006). A molecular phylogeny of the genus *Echinococcus* inferred from complete mitochondrial genomes. Parasitology.

[41] Thompson R.C.A., Lymbery A.J., Constantine C.C. (1995). Variation in *Echinococcus*: Towards a taxonomic revision of the genus. Adv. Parasitol..

